# Zinner Syndrome

**DOI:** 10.7759/cureus.31308

**Published:** 2022-11-09

**Authors:** Sachin Kumar, Kavin Ilangovan G, Alam Khalil-Khan, Alex Daniel P Arul Pitchai, Ramprasath Sathiamoorthy, Einstien Raju

**Affiliations:** 1 Department of Radiology, Chettinad Hospital and Research Institute, Chettinad Academy of Research and Education, Chennai, IND; 2 Department of Medicine and Surgery, Government Medical College, Omandurar, Chennai, IND; 3 Department of Academic Unit of Primary Medical Care, The University of Sheffield, Sheffield, GBR

**Keywords:** unilateral absence of the kidney, dysuria, wolffian duct anomaly, ejaculatory duct obstruction, cystic dilatation of seminal vesicle

## Abstract

Zinner syndrome is a less common birth anomaly of the Wolffian duct consisting of unilateral kidney absence, ipsilateral ejaculatory duct obstruction, and seminal vesicle cyst. A failure of embryogenesis of the ureteric bud between the fourth and 13th week of gestation results in Zinner syndrome. Conservative treatment is recommended for asymptomatic patients, whereas invasive treatment is reserved for symptomatic patients and for those who have failed conservative treatment. In this case report, we describe the non-specific presentation of lower abdominal pain and dysuria, as well as episodes of hematuria and new-onset hypertension, in a male patient, who was otherwise deemed healthy, with no other previous medical or surgical history. An imaging study and laboratory investigations were performed, and the patient was detected to have left renal agenesis and hypointense/hyperintense cysts in the left seminal vesicle of the left kidney. The findings supported the diagnosis of Zinner syndrome. The patient did not present with any symptoms or findings that would suggest infertility at the time of the study.

Zinner syndrome is a rare cause of painful micturition and hematuria in males and can be diagnosed using ultrasound (USS), computer tomography (CT), and magnetic resonance imaging (MRI) techniques. Zinner syndrome should be considered as a differential diagnosis in male patients with unilateral renal agenesis and cystic pelvic masses. Patients who are asymptomatic typically undergo conservative treatment and are followed up to prevent infertility. For patients with symptomatic cysts who fail to respond to conservative treatment or whose cysts are larger than 5 cm in diameter, surgical intervention is recommended (open or laparoscopic surgery and ejaculatory duct balloon dilatation).

## Introduction

Zinner syndrome is characterized by triple-featured manifestations, which include bilateral renal agenesis, ipsilateral obstruction of the ejaculatory duct, and cystic dilatation of seminal vesicles causing microcysts or macrocysts. The failure to meet metanephros is caused by the failure of ureteric buds to migrate from the mesonephric duct. Consequently, the metanephric blastoma fails to differentiate, which leads to ipsilateral renal agenesis and ejaculatory duct obstruction, which leads to cystic dilation of the ipsilateral seminal vesicle. Zinner syndrome occurs in just over 200 cases out of 100,000 patients [1]. It was recently reported that cyst-like lesions of the seminal vesicle are associated with the absence of kidneys on the ipsilateral side in males and that the incidence rate falls between 0.00035% and 0.0046% [2]. Typically, cystic lesions of the seminal vesicles develop in young male patients due to injury during embryogenesis, since acquired seminal vesicle cysts are rare and usually unilateral. The absence of symptoms leads to most patients being undiagnosed and being diagnosed incidentally during their second to fourth decade of life.

## Case presentation

**Figure 1:**
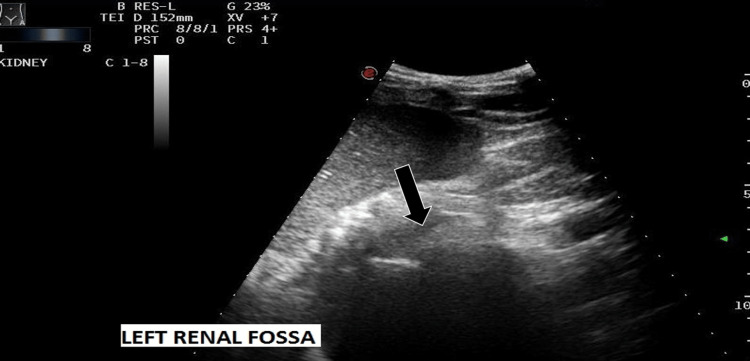
Abdominal ultrasound showing an absent left kidney in the left renal fossa (arrow)

**Figure 2:**
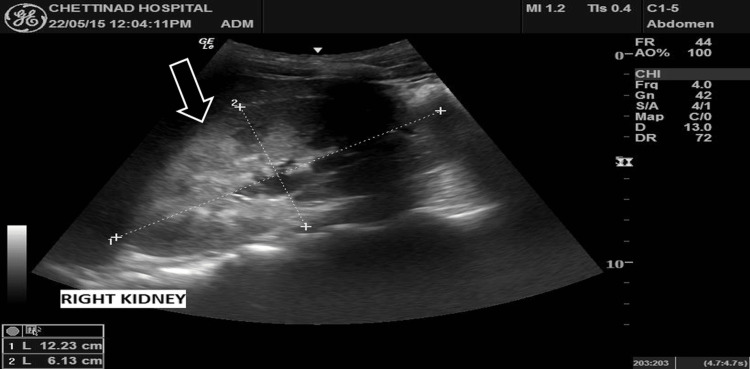
Mild compensatory hypertrophy of the right kidney (arrow)

**Figure 3:**
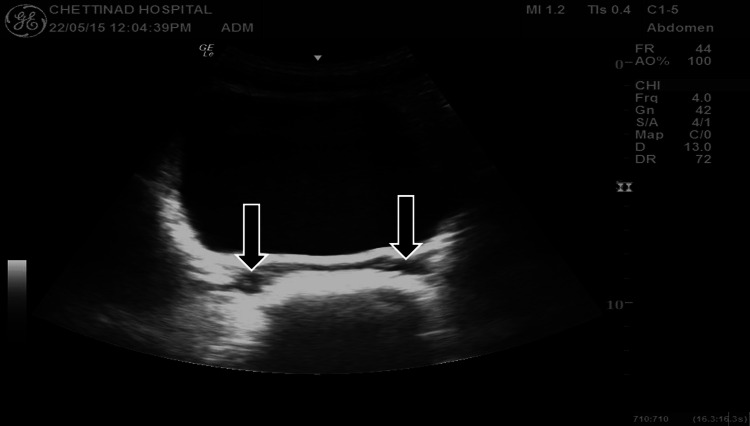
Ultrasound showing multiple tiny cystic lesions in bilateral seminal vesicles (arrows)

**Figure 4:**
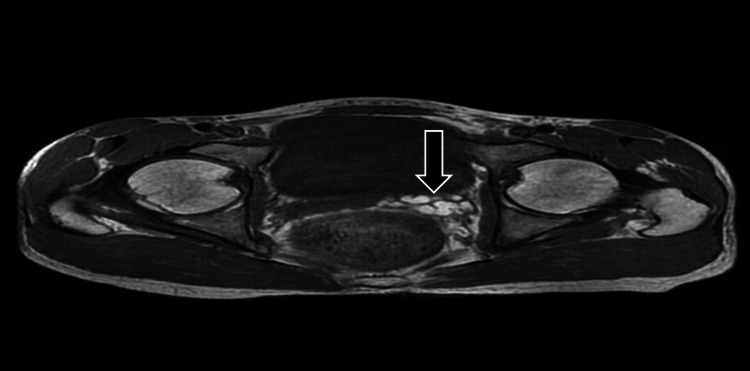
Magnetic resonance imaging (axial T1) showing hyperintense cysts on the left seminal vesicle likely to represent hemorrhagic changes (arrow)

**Figure 5:**
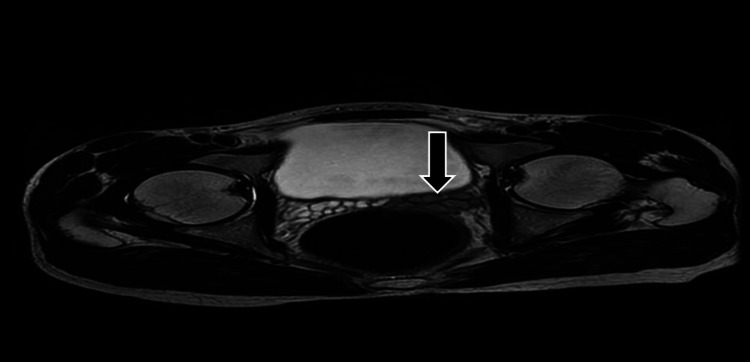
Magnetic resonance imaging (axial T2) showing hypointense cysts on left seminal vesicles (arrow)

**Figure 6:**
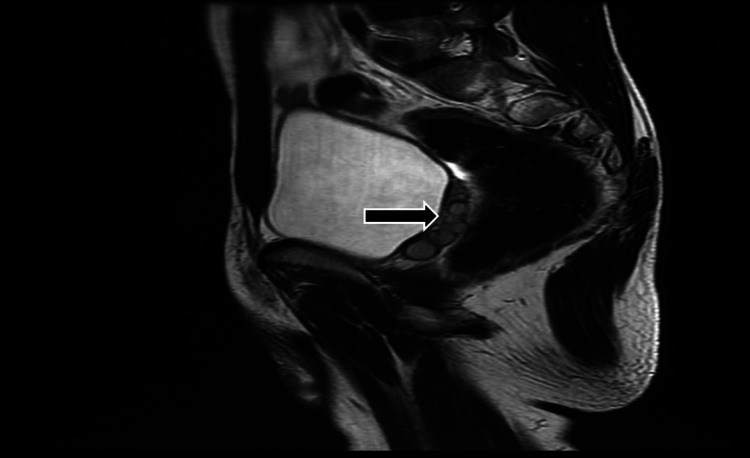
Magnetic resonance imaging (sagittal T2) showing hypointense left seminal vesicle cysts on T2 (arrow)

**Figure 7:**
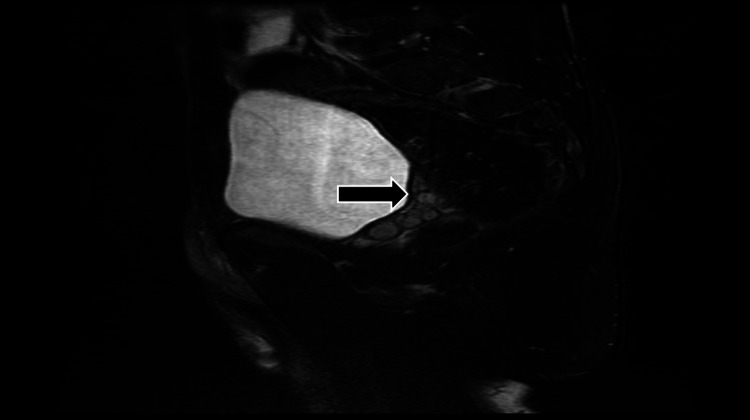
Magnetic resonance imaging (sagittal fat-saturated image) showing left seminal vesicle cysts with no evidence of suppression (arrow)

A 21-year-old male presented with a month history of lower abdominal pain coupled with dysuria and intermittent hematuria. The patient reported no abdominal trauma in addition to having no previous surgical or medical history, nor a history of infertility. Physical examination revealed new-onset hypertension (blood pressure (BP): 140/90), in addition to mild lower abdominal tenderness. Laboratory tests were requested and subsequently demonstrated a complete blood count, urea and creatinine, and liver function tests, which were all unremarkable. Urinalysis revealed no proteinuria with a few red blood cells (RBC). To date, the patient had not received any prior imaging studies to aid with comparison. Ultrasound (USS) of the abdomen demonstrated no visible left kidney in the renal fossa (Figure 1), no evidence of ectopic kidney, and mild compensatory hypertrophy of the right kidney (Figure 2), and multiple small cystic lesions were found within bilateral seminal vesicles (Figure 3). A transrectal USS was suggested to rule out an obstruction of the ejaculatory duct, but the patient declined. Further imaging studies were undertaken to rule out Zinner syndrome. Magnetic resonance Imaging (MRI) confirmed left renal agenesis and showed multiple small T2 hypointense/T1 hyperintense cysts within the left seminal vesicle (Figures 4-6), which are likely the result of hemorrhage. The cysts showed no evidence of signal loss on MRI fat saturation sequences (Figure 7).

In the right seminal vesicle, there were T2 hyperintense/T1 hypointense cysts with no diffusion restriction. Zinner syndrome was diagnosed based on the patient’s history, radiological findings, and clinical presentation. Analyses of the seminal fluid were reported as unremarkable. Subsequent treatment with antibiotics was commenced for an infection of the urinary tract. As the patient reported no infertility issues, with the addition of normal seminal fluid analysis, he was advised to undergo regular follow-ups. Following a two-week review, after the onset of the initial symptoms, the patient was symptom-free.

## Discussion

Most often, congenital abnormalities of the seminal vesicle are associated with abnormalities in the ipsilateral upper urinary tract, since both seminal vesicles and ureteral buds originate from the Wolffian duct, which is formed during the fourth week of pregnancy. Females with Mayer-Rokitansky-Küster-Hauser syndrome are considered to be the counterparts of males with Zinner syndrome [3]. Aside from the classical triad, it may also present with ureterocele, testicular or epididymis abnormalities, and hypospadias. Approximately 68% of the cystic dilation of seminal vesicles occurs when the kidney is absent ipsilaterally [2,4]. The mesoderm gives rise to the pronephros during the third week of life, which in turn forms the mesonephros by the end of the fourth week of pregnancy. A primitive kidney is formed by the ureteric bud, which arises from the lower end of the Wolffian duct in the fifth week [5,6]. Seminal vesicles arise from the distal Wolffian duct’s bulbous structure. During this period of embryogenesis, disruptions in any of these inductive events, such as mutations of metanephric blastema or disruptions in retinoic acid signaling, inhibit ureteric bud growth, failing to fuse with metanephric blastema, resulting in renal agenesis or hypoplasia [2,7]. The disruption of the distal segment of the Wolffian duct results in abnormalities of the ejaculatory duct and abnormal ureteric buds. An ectopic ureter can form when the ureteric bud arises distal to the urogenital sinus and may empty into seminal vesicle cysts, the vas deferens, the ejaculatory duct, or the bladder neck [2,7]. The obstruction of the ejaculatory duct can result in cysts in the seminal vesicles and dilated tubules. Zinner syndrome is therefore caused by abnormal embryogenesis of the ureteric bud.

Typical presentation includes abdominal pain, fullness, and symptoms related to micturition, such as obstructive urination, dysuria, urgency, and hematuria. Symptoms such as painful ejaculation [8], recurrent epididymitis, and infertility have also been reported. The majority of patients present in the second to fourth decade due to high levels of reproductive and sexual activity, but they may also present asymptomatically [2]. It is often misinterpreted due to its non-specific signs and lack of symptoms. Usually, microcysts measuring less than 5 cm in diameter are dormant in nature and are only detected incidentally. It is observed that patients with this condition usually present with obstruction of the bladder outlet, resulting in an increased incidence of infection within the urinary bladder, and that bladder compression can occur in the presence of cysts up to 12 cm in size [2]. Half of the reported cases of ejaculatory duct occlusion have been associated with a decreased amount of semen in both the quality and quantity aspects, as well as a decreased motility and sperm count. To determine if Zinner syndrome is associated with infertility, the seminal fluid must be examined. To identify pelvic masses, USS is the principal imaging modality, where seminal vesicle cysts are seen as anechoic thick-walled cysts on transrectal USS. There may be internal echos in a cyst as a result of infection or hemorrhage. To diagnose Zinner syndrome without requiring further invasive procedures, computer tomography (CT) and magnetic resonance imaging (MRI) are the most reliable methods. It is possible to detect renal agenesis and retrovesicular cystic pelvic masses as a result of enlarged seminal vesicles using a computed tomography scan. MRI aids in identifying seminal vesicle cysts and has a high signal intensity (SI) on T2 images and a low SI on T1 images. There can be a high signal intensity on T1 due to hemorrhage or the presence of proteinaceous fluid. Although malignant degeneration is extremely rare, intracystic vegetation may be an indication of malignancy. Seminal vesiculitis, ectopic ureteroceles, ejaculatory duct cysts (rarely associated with renal agenesis and are typically located midline), prostatic utricle cyst, other cystic lesions of the prostate, regional cystic neoplasm [9], and abscesses are among the differential diagnoses. A characteristic feature of Zinner syndrome is the absence of one kidney, occlusion of the ipsilateral ejaculatory duct, and the presence of microcysts in the seminal vesicles. In the literature, only a few cases of adenocarcinoma, cyst adenoma, and squamous cell carcinoma have been described in relation to microcysts of the seminal vesicle.

Management is determined depending on the size of the cysts, the symptoms of the patient, and the existence of complications. The prognosis is generally good in most cases. Surgery is the mainstay of management in symptomatic patients [10], patients who have failed conservative treatment, or if the cysts exceed 5 cm in size [2]. In the case of asymptomatic patients, conservative treatment with antibiotics is recommended. It is also possible to perform minimally invasive procedures such as transrectal ultrasonography-guided cyst aspiration.

## Conclusions

In males, Zinner syndrome causes painful micturition and hematuria. Most patients are asymptomatic and are diagnosed with imaging modalities or in later stages of their life. Thus, Zinner syndrome should be considered as a differential diagnosis in cases of unilateral renal agenesis in males with cystic pelvic masses. Today, a wide variety of imaging modalities help to identify and differentiate between pelvic masses. While abdominal and transrectal USS can be helpful, only CT and MRI can provide accurate diagnoses of Zinner syndrome. It is important to follow up with asymptomatic patients to prevent infertility and determine the course of treatment.
